# Factors associated with HIV voluntary disclosure of people living with HIV to their steady sexual partner in the Democratic Republic of the Congo: results from a community-based participatory research

**DOI:** 10.11604/pamj.2014.19.276.5304

**Published:** 2014-11-14

**Authors:** Eddy Kieto Zola, Gaspard Matamba Gifudu, Emilie Henry, Adeline Bernier, Henri Mukumbi Masangu, Alise Abadie, Lionel Fugon, Joanne Otis, Marie Préau

**Affiliations:** 1 Ecole de Santé Publique de l'Université de Kinshasa, République Démocratique du Congo; 2 Actions Communautaires Sida/Avenir Meilleur pour les Orphelins au Congo, République Démocratique du Congo; 3 Coalition Internationale Sida, Tour Essor, 14 rue Scandicci, 93500 Pantin, France; 4 Université du Québec à Montréal, CREcES, Montréal, Canada; 5 GRePS, Institut de Psychologie, Université Lumière Lyon 2, Lyon, France

**Keywords:** HIV, serostatus disclosure, sexual partner, testing, community-based research

## Abstract

**Introduction:**

HIV disclosure to a steady sexual partner (SSP) is important both in preventing HIV transmission and improving the quality of life of people living with HIV (PLHIV). Its determinants have been poorly investigated in the Democratic Republic of the Congo. The study objective was to determine factors independently associated with voluntary disclosure to one's SSP in PLHIV receiving services from a Congolese community-based organization (CBO).

**Methods:**

A community-based participatory research was performed and 300 PLHIV were interviewed by members of the CBO, using a standardized questionnaire. A multivariate logistic regression was used to determine the variables independently associated with disclosure.

**Results:**

In this sample, 79 of the 127 participants (62%) included in the analysis declared having voluntarily disclosed their serostatus to their SSP. Declaring to be in a relationship (Odds Ratio (95% Confidence Interval): 4.2 (1.4-12.6)), having tested for HIV because of symptoms (2.5 (1.0-6.4)), having taken the test on one's own initiative (3.2 (1.3-8.0)), having felt sympathy and indifference from people when disclosing (6.0 (1.4-26.9) and 5.0 (1.1-22.8), respectively) as well as having a higher score of the “regular discussion about daily life with HIV” index (1.7 (1.1-2.5)) were significantly associated with disclosure to one's SSP.

**Conclusion:**

Several individual and contextual factors were associated with voluntary disclosure to SSP in this study, highlighting the complex nature of the disclosure process. Interventions encouraging disclosure should be designed so as to adapt to one's personal life with HIV as well as psychosocial environment.

## Introduction

Newly diagnosed HIV positive people are all confronted with the issue of “disclosure”, that is to say the opportunity to inform-on one's own initiative- a family member, a friend or a sexual partner about their seropositivity.

The WHO encourages health professionals to tackle the question of disclosure both during HIV pre-test counseling and after the diagnosis [1]. Many care providers promote the advantages of disclosure to the individual, especially the possibility of receiving moral and material support from their partner, family and friends [2–4]. From a public health perspective, disclosure to (a) sexual partner(s) entails the possibility for the couple to discuss strategies on how to reduce the risk of infection, in particular by increasing condom use [5–7]. Disclosure has been linked to earlier testing of the sexual partner and access to care, if necessary [8, 9]. However, negative aspects of disclosure have been highlighted by many studies. First, disclosure does not systematically result in a reduction of risky practices in serodiscordant couples [10–12]. Second, the risk of stigmatization and discrimination which the individual is exposed to after disclosure is still very strong [13]. The reasons most frequently cited by PLHIV for not disclosing their serostatus are the risk of stigmatization and discrimination, as well as the risk of their relationship breaking up, of being abandoned, of divorce and of violence [3, 14–16]. Negative reactions of the steady sexual partner (SSP) after disclosure can have dramatic consequences for the PLHIV. Consequently, PLHIV face a dilemma when willing to disclose, anticipating both positive and negative reactions.

Numerous factors have been associated with disclosure: at the individual level, sociocultural factors, religious beliefs and social representations; at the structural level, the epidemiological context, the organization of the healthcare system and the conditions of access to testing and care [8, 17–23]. In the Democratic Republic of the Congo (DRC), where the act of not disclosing one′s serostatus to one′s partner is a felony since 2008, the disclosure process and the factors associated with disclosure have been poorly investigated so far. Considering the sensitive nature of this issue, the importance of community mobilization in the issue of disclosure, and in the framework of our ethical considerations, we conducted a community-based study to determine the factors associated with HIV voluntary disclosure to one′s steady sexual partner (SSP) of PLHIV in contact with a community-based organization (CBO) in the DRC.

## Methods

***“Partages” project:*** this study is a sub-study of a community-based cross-sectional research project, “Partages” (which means disclosure in French). Developed and implemented by a mixed (researchers/CBO members) international research consortium from seven countries (Canada, the DRC, Ecuador, France, Mali, Morocco and Romania), its objective was to document the factors associated with serostatus disclosure by PLHIV in contexts where available data are rare, using community-based participatory research (CBPR) principles [24, 25].

CBPR is a form of research aiming at directly involving members of the community affected by the topic being investigated, using a collaborative approach involving academic researchers and community stakeholders in an equitable partnership ensuring mutual benefits. Community members and researchers are involved in each stage of the research process, from designing the research question to disseminating the results. In Africa, this type of research has long been in existence, allowing the gradual emergence of a participatory health democracy [24]. This form of research is now increasingly used and participates in the global movement of community mobilization, empowerment and representation.

In the “Partages” project, CBPR principles have been adhered to throughout the process. CBOs members, PLHIV and researchers were involved, in an equitable partnership. Tools, like a memorandum of understanding, were developed before the start of the project to ensure mutual respect and understanding, balanced power in the decision-making as well as shared control over all phases of the research process. Community members were trained to research methods and research ethics. The results of the project were presented to participants and stakeholders in all the countries where data were collected. Community mobilization was strong during the whole process. This project gave a voice to the community about a very sensitive issue.

***Participants:*** the inclusion criteria were as follows: being HIV positive, being 18 year-old and over, and being aware of one's seropositivity for more than six months. In total, between May and October 2011, 1500 participants were recruited in five countries (the DRC, Ecuador, Mali, Morocco and Romania) (300 per country). In the DRC, a convenience sample of 300 PLHIV who were in contact with the CBO ACS-AMO Congo in Kinshasa was recruited at two outpatient treatment centers, one in Kasa-Vubu and the other in N'Djili. PLHIV were asked during their routine medical visit at one of these sites if they were willing to participate to the study. As the study was cross-sectional and the study participants were already “in site” for their medical visit, they did not receive reimbursement for transportation. Kinshasa′s School of Public Health ethical committee approved the study in February 2011.

***Questionnaire and procedure:*** after providing written informed consent, participants were interviewed face-to-face by CBO members, most of whom were members of ACS-AMO Congo living with HIV. All were trained beforehand in interviewing techniques. The questionnaire was developed by the whole team, then tested on the field by CBO members and PLHIV. The questionnaire included 125 questions, divided into eight sections: socioeconomic data, history and contact with HIV, serostatus disclosure and reaction of others to disclosure, self-efficacy, intimate and social lives, sexuality, quality of life and contact with CBOs [26]. The same questionnaire was used in the five countries where data were collected. Data were kept strictly confidential. CBO beneficiaries were assured that they would continue to receive all the services offered by ACS-AMO Congo, independently of whether they accepted or refused to participate in the study or whether they interrupted participation.

***Voluntary disclosure to one′s steady sexual partner:*** herein, we analyzed the answer for the DRC participants to the following question: “Have you disclosed your seropositivity to your steady sexual partner?”. The possible answers were “No”, “Yes”, or “I don′t have a steady sexual partner”. Only participants who declared having a steady sexual partner and who answered either “Yes” or “No” to this question were included in the analysis.

***Explanatory variables:*** based on existing literature and field experience of community members, the following variables were tested for their association with voluntary disclosure to the SSP: gender, age, relationship status, having children, main activity, number of years since HIV diagnosis, circumstances of HIV test and origin of initiative, reactions to disclosure, need to discuss HIV with healthcare providers and cessation of sexual relations because of HIV seropositivity. We also included in the analysis an index - hereinafter referred to as the “regular discussion about daily life with HIV" index-, built using the following questions: “Which of the following people do you regularly speak to about your seropositivity / your daily life with HIV? ”. Binary answers for the following categories were summed: other PLHIV, family members, friends, doctors/people providing medical support and care, and members of the CBO. The more the participant regularly discussed these issues with people from different groups, the higher the index score (the maximum being 5).

***Statistical analysis:*** the sample was weighted using a variable based on the sociodemographic characteristics (age group, gender and recruitment site) of PLHIV followed by ACS-AMO Congo, to ensure that the sample was representative of the population followed by the CBO. As mentioned previously, only participants who answered “Yes” or “No” to the question about disclosing seropositivity to one′s SSP were included in the analysis. Categorical variables were compared using Chi-2 test or Fisher exact test, and for continuous variables, the comparisons of the means were performed using Student t-test or non parametric tests (Kruskall-Wallis or Wilcoxon test). Potential explanatory variables were screened by testing each one independently for an association with HIV voluntary disclosure to one's SSP, using weighted univariate logistic regression. Variables that achieved a significance level of p ≤ 0.20 in the univariate analysis were included in the multivariate analysis. For the multivariate analysis, a complete case analysis was performed and the final model was built using a backward elimination approach based on the log-likelihood ratio test (p ≤ 0.05). A receiver operating characteristic (ROC) curve was used to assess the logistic regression model's ability to accurately distinguish individuals who had disclosed from the others. The area under the ROC curve (AUC) provided a measure of discrimination [27]. Continuous variables are presented as mean ± standard deviation. Data management and statistical analyses were performed using SPSS v20.0 (IBM Corp., 2011). Statistical analysis was also performed in line with the CBPR approach. For this analysis, the person in charge went to the DRC, organized meetings to identify and design the research question with community stakeholders and local researchers. The model was built in an iterative process, with contributions of community stakeholders and researchers at every step, from the univariate analysis to the results interpretation.

## Results

Among the 300 participants, 127 people declared having a SSP and answered the questionnaire item related to HIV voluntary disclosure to a SSP. Among them, 79 (62%) declared that they had voluntarily disclosed their seropositivity to their SSP. A description of the characteristics of these 127 participants is presented in Table 1.

**Table 1:** Characteristics of participants included in the analysis (n = 127)

Variables	Categories	Total sample (n = 127) n (%) or mean (SD)	Participants who disclosed their serostatus to their SSP (n = 79)) n (%) or mean (SD)	Participants who did not disclose their serostatus to their SSP (n = 48)) n (%) or mean (SD)
Gender	Man	57 (45)	38 (48)	19 (40)
	Woman	70 (55)	41 (52)	29 (60)
Age		44.3 (10.5)	44.2 (8.5)	44.4 (13.3)
Current relationship status	Not in a relationship	24 (19)	8 (10)	16 (33)
	In a relationship	103 (81)	71 (90)	32 (67)
Having children	No	11 (9)	3 (4)	8 (17)
	Yes	116 (91)	76 (96)	40 (83)
Main activity	Unemployed / Housewife / Student	38 (30)	23 (29)	15 (31)
	Formal or informal employment	89 (70)	56 (71)	33 (69)
Number of years since HIV diagnosis		3.6 (2.9)	3.9 (3.1)	3.1 (2.5)
HIV test because of symptoms	No	40 (31)	20 (25)	20 (42)
	Yes	87 (69)	59 (75)	28 (58)
Test on PLHIV's own initiative	No	71 (56)	37 (47)	34 (71)
	Yes	56 (44)	42 (53)	14 (29)
Reaction after disclosure : sympathy/support	No	16 (13)	7 (9)	9 (19)
	Yes	111 (87)	72 (91)	39 (81)
Reaction after disclosure : indifference/denial	No	105 (83)	61 (77)	44 (92)
	Yes	20 (16)	16 (20)	4 (8)
“Regular discussion about daily life with HIV” Index		2.9 (1.1)	3.1 (1.1)	2.4 (1.0)
Need to discuss HIV with healthcare providers	No	37 (29)	17 (21)	20 (42)
	Yes	89 (70)	61 (77)	28 (58)
Cessation of sexual relations because of HIV seropositivity	No	101 (80)	68 (86)	33 (69)
	Yes	26 (20)	11 (14)	15 (31)

SD: Standard Deviation

There were slightly more women than men (55%) and mean age was 44.3 years. More than eight people in 10 (81%) declared that they were in a relationship (married or not), and the vast majority of the sample (91%) had children. Most of them were employed (70%), either formally or informally. The number of years since HIV diagnosis was 3.6 years, on average. More than two-thirds of the participants (69%) performed the test because of symptoms, and less than one in two on their own initiative. Reactions after disclosure were mostly positive, as 87% of them experienced reactions of sympathy and support. A vast majority of participants (70%) felt the need to discuss HIV with healthcare providers. Finally, 20% of participants ceased having sexual relations with their partner because of HIV seropositivity.

In Table 2, results of multivariate analysis are described. Multivariate analysis identified a positive, independent and statistically significant association of the following factors with HIV voluntary disclosure to one's SSP: declaring to be in a relationship (Odds Ratio (95% Confidence Interval): 4.2 (1.4-12.6), p = 0.01), having been tested for HIV after the onset of symptoms (2.5 (1.0-6.4), p = 0.05), having been tested on one's own initiative (3.2 (1.3-8.0), p = 0.01), having felt sympathy/support when disclosing serostatus (6.0 (1.4-26.9), p = 0.02), having felt indifference/denial when disclosing serostatus (5.0 (1.1-22.8), p = 0.04), and having a higher “regular discussion about daily life with HIV” index score (1.7 (1.1-2.5), p = 0.01). The AUC was 0.78, indicating an acceptable degree of discrimination according to Hosmer and Lemeshow [27].

**Table 2:** Factors significantly associated with serostatus disclosure to one's steady sexual partner in multivariate analysis (n = 126)*

Variables	Categories	aOR [95% CI]	P-value
Current relationship status	Not in a relationship	1	0.01
	In a relationship	4.2[1.4–12.6]	
HIV test because of symptoms	No	1	0.05
	Yes	2.5[1.0–6.4]	
Test on PLHIV's own initiative	No	1	0.01
	Yes	3.2[1.3–8.0]	
Reaction after disclosure: sympathy/support	No	1	0.02
	Yes	6.0[1.4–26.9]	
Reaction after disclosure: indifference/denial	No	1	0.04
	Yes	5.0[1.1–22.8]	
“Regular Discussion about daily life with HIV” Index		1.7[1.1–2.5]	0.01

aOR = adjusted Odds Ratio; CI = Confidence Interval

* As a complete case analysis was performed to build the multivariate model, one participant was excluded from the analysis because of a missing value for the variable “Reaction after disclosure: indifference/denial”

## Discussion

In this study, 62% of the participants declared that they had voluntarily disclosed their serostatus to their SSP. This is very similar to the 59% reported in a recent study in the DRC [28]. In other studies, the disclosure rate to one′s SSP varies between <20% and >90% [3, 6, 23, 29, 30]. These variations are dependent on study design, but they also show the importance of exploring contextual and individual determinants of disclosure to one's SSP [31].

Our results showed that declaring being in a relationship, whether married or not, was positively associated with disclosure to one's SSP. The association between being married and disclosure to one's steady partner has been highlighted in several studies, especially in women [8, 29]. Declaring oneself to be in a relationship may be a sign of affective engagement with one's partner and trust. Several studies showed that being in an emotional relationship with a partner increased the feeling of responsibility to disclose to him/her, feeling which arise from the PLHIV's sense of obligation to be transparent, honest, and even “morally obliged” when it comes to disclosure [8, 12, 32–34]. Disclosure may also reflect the PLHIV's concern to limit the risk of transmission to his/her partner, or to encourage the latter to go and have a HIV test. Hoping that the partner will react positively, because of the intimacy of their relationship, may also encourage PLHIV to disclose to him/her, in order to obtain moral and/or financial support. The study by Desgrées-du-Loû, which focused on the marital/relationship consequences of pre-natal HIV testing in Abidjan, showed that the reaction of males to their female partner's disclosure of seropositivity greatly depended on the pre-existing nature of the partners’ relationship [35]. Deribe *et al*. also highlighted the association, in women, between disclosure and perceiving one's relationship as a lasting one [29]. Trust in the partner's capacity to keep the PLHIV's seropositivity confidential, in order to protect the household, may limit worries associated with disclosure and facilitate the decision [36]. In our study, the majority of people who declared that they were married or in a couple lived with their partner. Yaméogo *et al*. found that the rate of disclosure to one's partner was particularly high amongst “cohabiting couples” and identified an association between cohabitation and a trusting relationship, where shared confidentiality between the two partners is facilitated [8]. For a person living in a couple and taking treatment, trying to keep his/her serostatus confidential may make adherence difficult, as this entails hiding oneself to take pills at specific moments of the day [37]. Being able to openly and correctly take one's medication, and even to be accompanied when going to pick up one's anti-retrovirals prescription, or having someone go to pick up the prescription for the PLHIV when the latter cannot do so are several possible motivations for disclosure to one's SSP when living together [38].

In our study, a link was found between certain circumstances surrounding diagnosis and disclosure to one's SSP. Sixty nine percent of our study respondents declared that their HIV test was linked to the onset of symptoms. A positive association was found between having been tested because of symptoms requiring medical attention and disclosure to one ‘s SSP. This association may be explained by the fact that a person who has already developed symptoms directly or indirectly associated with HIV infection may be at a more advanced stage of the disease or have such a poor state of health that it becomes much more difficult to keep his/her seropositivity a secret. Indeed, several studies showed a link between disease progression and disclosure, in particular disclosure to a sexual partner [16, 39–41]. These results also suggest a late access to HIV testing and care in the DRC with negative effects on the health and psychological well-being of PLHIV.

Having been tested on one's own initiative was also positively associated with HIV voluntary disclosure to one's SSP in our study. A person who decides him/herself to have a HIV test may be more prepared for a possible positive diagnosis. Kadowa and Nuwaha's study showed that people who went to be tested in voluntary testing and counseling centers had thought about having the test for a longer time [14]. A person who decides on his/her own initiative to be tested and who is subsequently diagnosed positive may be more psychologically prepared to deal with the consequences of his/her seropositivity, including the reactions of close family, friends and sexual partner(s) to the PLHIV's announcement of his/her serostatus. Rutledge showed that in order for PLHIV to be able to confide in and disclose their seropositivity to their sexual partners, they had to already have psychologically accepted their infection, the change in identity arising from the infection, and the associated responsibilities in terms of sexual relations [33].

Feeling either sympathy/support and/or indifference/denial from a confidante when seropositivity was disclosed was also found to be positively associated with disclosure to one's SSP. This association may be interpreted in two ways: a PLHIV who has already experienced those reactions to disclosure may feel more confident about the possibility that his/her partner will have a positive or neutral reaction. Indeed, Simbayi *et al*. showed that not having discussed one's seropositivity with one's friends and not having disclosed it to others for fear of a negative reaction was associated with non-disclosure to sexual partners [42]. Another interpretation would be that if a PLHIV started the disclosure process with his/her SSP and that he/she had a positive reaction, it might bring strong moral support for the PLHIV to accept his/her serostatus and give self-confidence to disclose to other people under proper conditions, which in turn may increase the probability that disclosure targets react positively or neutrally to the announcement.

Finally, regularly discussing one's seropositivity with more categories of persons, as other PLHIV, family members, friends, doctors, counselors and CBO members was positively associated with disclosure to one's SSP. Previous studies suggested that personal communication skills and communication patterns are important determinants of disclosure and that people who declared having better communication skills were more likely to disclose their serostatus [14, 43]. Improving interpersonal communication skills in terms of HIV appears to be a necessity for disclosure [44]. When an environment exists (be it CBO, family, friend or work-based) which encourages discussion about his/her seropositivity, disclosure to a SSP may appear less daunting for the PLHIV, as he/she can count on a strong support network to overcome what is often a difficult ordeal [45]. The support PLHIV receive from other people strengthens their self-confidence in their capacity both to get others to accept the disease and to obtain more support. Consequently, this may facilitate disclosure to their partner.

In addition to the results of this study, the process in itself was important. The CBPR approach empowered community stakeholders and PLHIV in five different countries. Strong partnerships were created throughout the process between researchers and community members. Several workshops were organized with researchers and community members from the seven countries of the consortium, allowing fruitful exchanges of experience and mutual empowerment. And social change, which is a goal in CBPR, was achieved, as the management of the serostatus disclosure issue changed after the project among CBO members and community leaders.

**Limitations:** our study has some limitations. First, recruitment was done using a convenience sample of PLHIV in contact with a local CBO working in the fight against HIV. The fact that the PLHIV came regularly to the CBO's facility for care and that they trusted the organization's ethical code facilitated rapid and quality-ensured recruitment for the study. Nevertheless, the beneficiaries of this organization's services are supported medically, psychologically and socially. In particular, they are encouraged and supported in their decision to disclose their serostatus. Therefore, PLHIV in this sample might have disclosed more than those who are not in contact with CBOs [46]. This sample then cannot be considered as representative of all the PLHIV in the DRC. Second, the limited number of participants in this study might have been a limiting factor to analyze factors associated with voluntary disclosure. Indeed, confidence intervals are very wide in the multivariate analysis. Third, the study used a cross-sectional design, therefore limiting the capacity of the study to capture the dynamics of the disclosure process. Finally, the existence of a law criminalizing non-disclosure to sexual partners in the DRC could have introduced a selection and/or a desirability bias. Nevertheless, this bias should be very limited since the law has never been enforced in the DRC. Most PLHIV did not even know its existence. Moreover, the fact that the study was conducted by NGO members who had been trained on the study protocol and ethical issues as well as the pre-existing confidence relationship between respondents was rather a guarantee of good quality answers.

## Conclusion

This community-based study highlighted several types of factors associated with serostatus disclosure to one's SSP, underlining the importance of the marital and affective situation, the circumstances of the HIV test as well as the social context and environment in which PLHIV live. These results suggest that multi-level interventions are needed to facilitate serostatus disclosure to one′s SSP. On the field, CBOs could develop interventions to reinforce PLHIV′s communication skills and to design individualized support taking into account personal experience and environment. At the structural level, there is a need to reinforce the fight against stigmatization in the general population, so as to create a secure psychosocial environment for PLHIV ensuring positive reactions and support of their families and friends if the PLHIV is willing to disclose. Finally, this study further illustrates the advantages of performing a community-based participatory research when dealing with a sensitive topic.
